# Improving patient safety during position changes under general anaesthesia: “CHOPP check”- a new patient safety and educational tool

**DOI:** 10.12669/pjms.39.4.7965

**Published:** 2023

**Authors:** Kareem Hussein, Shankar Lal, Michael Moore, Irene Leonard

**Affiliations:** 1 Kareem Hussein, Specialist Registrar, Anaesthesiology and Intensive Care Medicine, Department of Anaesthesiology and National Neurosurgical Centre, Beaumont Hospital, Dublin, Ireland; 2 Shankar Lal, Fellow, Neuroanaesthesia and Neurocritical Care Medicine, Department of Anaesthesiology and National Neurosurgical Centre, Beaumont Hospital, Dublin, Ireland; 3 Michael Moore, Clinical Associate Professor, Anaesthesiology, Department of Anaesthesiology and National Neurosurgical Centre, Beaumont Hospital, Dublin, Ireland; 4 Irene Leonard Clinical Associate Professor, Anaesthesiology, Department of Anaesthesiology and National Neurosurgical Centre, Beaumont Hospital, Dublin, Ireland

**Keywords:** Patient positioning, Neurosurgical procedures, General anaesthesia, Complications

## Abstract

The requirement to change position whilst under general anaesthesia may expose patients to significant risks. We devised and implemented a concise and comprehensive patient positioning safety tool with the aim of reducing risks and improving patient safety during position changes under anaesthesia.

Optimal patient positioning during neurosurgical procedures is essential in ensuring adequate surgical access to pathological lesions and optimal intracranial pressure and cerebral blood flow to facilitate safe surgical resection. Depending on the site of the lesion and the surgical approach, patients may need to be placed in either the supine, lateral, prone or sitting positions. Anaesthesia is usually induced with the patient in the supine position, and after induction of general anaesthesia, the patient is then moved into the required position for surgery.

Changing position whilst under general anaesthesia may expose patients to many potential risks including, but not limited to, physical injury, interruption of essential monitoring, airway compromise, haemodynamic instability, impairment of ventilation and dislodgement of intravascular access and haemodynamic monitoring lines. Serious adverse effects on pulmonary physiology may occur, including a reduction in functional residual capacity and ventilation-perfusion mismatch leading to hypoxaemia.

Adverse haemodynamic effects, including reduced venous return, increased intra-thoracic pressure and reduced systemic vascular resistance, may result in significant hypotension, which could potentially compromise organ perfusion resulting in serious complications and adverse patient outcomes. These effects are more likely to be exaggerated in those with pre-existing comorbidities such as obesity and chronic cardiac and pulmonary disease. According to the anaesthesia closed claims project spanning from 2000 to 2014, it has been revealed that spine surgery claims accounted for more than 10% of the total claims. Furthermore, some of these claims were linked with severe permanent disability, mainly resulting from nerve and eye injuries.^1^ Safety checklists have become an established tool for improving patient safety and reducing the incidence of adverse events in medical practice.^2^


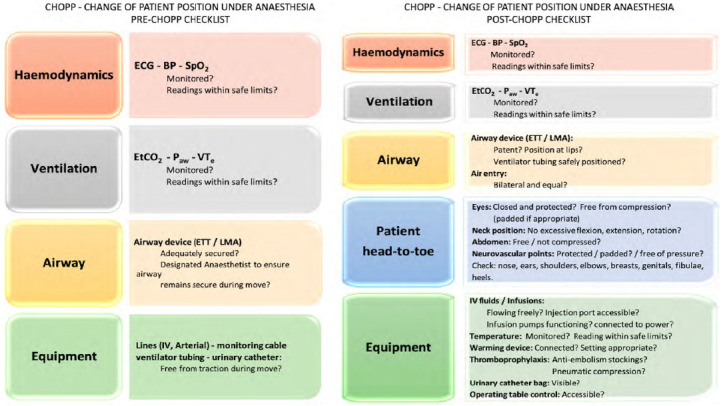


## Aim:

- Reduce risk and improve patient safety during changes of patient position under general anaesthesia.
- Develop a new practical educational tool for working anaesthesia and neurosurgery staff to ensure safe and confident clinical practice when managing patient position changes under anaesthesia.

## METHODS

After observation of existing operating team practices for several weeks, we identified key patient safety risks and key times of most significant risk during patient position changes under anaesthesia in our neurosurgical operating rooms. We then devised new pre- and post-position change safety checklists and introduced a brief safety pause to facilitate their completion.

To enhance user-friendliness, we developed the acronym “**CHOPP check” CH**ange-**O**f-**P**atient-**P**osition under anaesthesia **Check**list.

CHOPP-check comprises safety checks at two-time points: a) **before** and b) **after a** **change of patient position** under anaesthesia. It focuses on four key domains of potential patient risks: haemodynamic stability, adequacy of ventilation, patient head-to-toe check and equipment function. Important considerations in design were that CHOPP-check is:

- ***Comprehensive:*** Incorporates a check on all patient safety risks we had identified during our period of observation of position changes.
- ***User-friendly:*** a) can be completed in 1-2 minutes and b) works with the ergonomics of the operating room and can be completed from a single standing position with only the patient “head-to-toe” check requiring movement around the operating room.
- ***Has the potential for application:*** To other surgical specialities for any procedure which requires a patient position change under anaesthesia.

Staff education was delivered to an initial pilot group. Laminated checklists were created and displayed in prominent locations at key sites. Practice on-site drills, in addition to on-site simulation training of key operating room staff, were undertaken prior to roll-out.


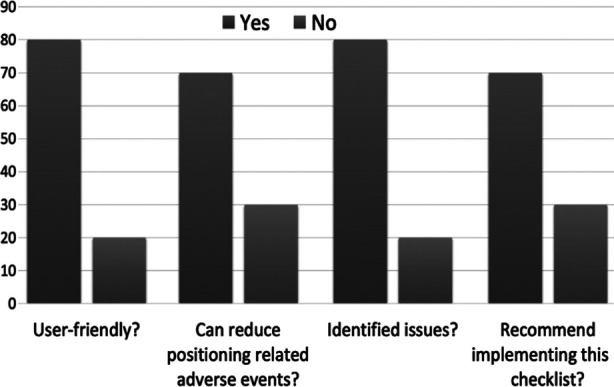


## RESULTS

After the ‘*CHOPP-check’* roll-out, staff surveys were undertaken to assess users’ perception of usefulness and impact on risk reduction. 80% of the respondents found CHOPP check to be user-friendly and supported its implementation. About 70% of *CHOPP checks* performed identified potential patient safety issues. Common issues identified were traction with potential or actual disconnection of monitoring cables and vascular access lines during position changes. Less frequently, more serious, potential airway incidents were identified. Support was high amongst surgical and anaesthesia staff for the implementation of this new safety tool.

**CHOPP-check** is a new patient safety tool that focuses staff attention on potential hazards at times of increased risk during patient position changes under anaesthesia. It provides a concise and practical tool to guide operating theatre staff during position changes and enhances their ability to reduce risk and improve patient safety. It has the potential for application to other surgical and non-surgical specialities in which procedures require changes of patient position under anaesthesia.

### Authors’ contributions:

**KH** and **SL:** Initial write-up.

**MM** and **IL:** Final write-up.

All authors reviewed the manuscript before submission.
